# Case report: Novel three-dimensional echocardiographic methods mapping aortic root pseudoaneurysm secondary to blood culture-negative endocarditis with bicuspid aortic valve involvement

**DOI:** 10.3389/fcvm.2023.1138390

**Published:** 2023-03-16

**Authors:** Yue Miao, Yanchao Zhang, Ling Yue, Shuang Guan, Wei Feng

**Affiliations:** Department of Ultrasound, The Fourth Hospital of China Medical University, Shenyang, China

**Keywords:** infective endocarditis, blood culture-negative endocarditis, pseudoaneurysm, three-dimensional echocardiography, TrueVue, TrueVue Glass

## Abstract

**Background:**

Infective endocarditis (IE), though uncommon, is a potentially lethal disease. Blood culture-negative endocarditis (BCNIE) accounts for 2.5%–31% of all cases of IE and can lead to life-threatening complications, including aortic root pseudoaneurysm. It is associated with considerable diagnostic and therapeutic dilemmas. TrueVue and TrueVue Glass include the latest two technologies applied in advanced three-dimensional echocardiography, which allow for novel photorealistic images of cardiac structures, and provide abundant previously unavailable diagnostic information. Herein, based on a series of novel three-dimensional echocardiographic methods, we report a case of BCNIE with aortic valve involvement, leading to aortic valve perforation and prolapse, and developing into a giant aortic root pseudoaneurysm.

**Case summary:**

In this study, we presented a case of a 64-year-old man exhibiting symptoms of intermittent fever, asthenia, and dyspnea following light exertion. Physical examination, laboratory tests, and electrocardiograms were suspected of IE, though the results of blood cultures were exactly negative. Three-dimensional transthoracic echocardiography, as well as a series of novel advanced techniques, was adopted to clearly visualize the lesions of the aortic valve and aortic root. However, despite active medical treatment modalities, the patient eventually suffered from a sudden, unexpected death 5 days later.

**Conclusion:**

BCNIE with aortic valve involvement and development into a giant aortic root pseudoaneurysm is a rare and serious clinical event. In addition, TrueVue and TrueVue Glass offer unprecedented photographic stereoscopic images, enhancing the diagnostic performance of such structural heart diseases.

## Introduction

Infective endocarditis (IE), though uncommon, is a substantial cause of morbidity with an annual incidence of 3–10/100,000 cases, leading to a huge burden on society worldwide (1). Blood culture-negative endocarditis (BCNIE) accounts for 2.5%–31% of all cases of endocarditis, which is often accompanied by considerable diagnostic and therapeutic dilemmas (2). Additionally, peri-annular spread of endocarditis contributes to tissue necrosis and peri-annular abscess, and in rare cases, precipitates pseudoaneurysm, which is a life-threatening complication (3). Currently, there is a lack of reports concerning patients with BCNIE with aortic valve involvement and development of a giant aortic root pseudoaneurysm; its early identification and accurate diagnosis remain formidable clinical challenges.

Advances in echocardiography techniques over the past few years have played an integral and vital role in the diagnosis of IE. Recent enhancements in three-dimensional echocardiography (3DE) have been further improved as a result of novel transducers, software, and trans-illumination techniques, which provide plentiful and previously unavailable diagnostic information. Meanwhile, the TrueVue technique allows for novel photorealistic images of cardiac structures with a freely movable virtual light source integrated into the data set, effectively enhancing the depth perception and augmenting the visualization of anatomical structures (4). In addition, the TrueVue technology can be modified into a transparent mode, named TrueVue Glass, focusing on the blood pool–tissue interface; however, the tissue itself remains transparent, which leads to highlighted borders of the chambers, valves, and vessels within the heart (5).

Herein, in the current study, we present a case suspected of BCNIE with aortic valve involvement, causing aortic valve perforation, and prolapse, and development into a giant aortic root pseudoaneurysm with novel 3DE imaging technologies.

## Case description

The included case concerned a 64-year-old man who presented with a medical history of diarrhea and intermittent low-grade fever for 2 months. The patient received repeated, inadequate antibiotic treatments at other medical institutions prior to admittance to our Department of Cardiovascular Disease. The patient arrived at our Department of Cardiovascular Disease for further diagnostics because of the worsened symptoms, including dyspnea following light exertion, asthenia, and more frequent bouts of fever in the last few days.

During physical examination, the patient was conscious and responded well, in addition to having an anemic appearance and a fever of 37.5°C. A Levine's grading scale II/VI systolic blowing murmur was auscultated in the apex area. Additionally, a diastolic murmur could be heard in the second auscultation area of aortic valve. Breath sounds in both lungs were rough. In addition, the patient's heart rate was 80 beats/min, blood pressure was 120/60 mmHg, and respiratory rate was 18 breaths/min. Laboratory tests illustrated an increase in the white blood cell count (12.90 × 10^3^/μL), B-type natriuretic peptide (1,367.38 pg/mL), C reactive protein (99 mg/L), and procalcitonin (0.155 mg/dL). The patient was mildly anemic with a hemoglobin level of 90 g/L. Moreover, the levels of antistreptolysin O and rheumatoid factors were both normal. Biochemical tests for antinuclear antibodies and functional tests for the thyroid were negative. Interestingly, three consecutive sets of blood cultures were all found to be negative. The thoraco-abdominal computed tomography (CT) revealed the presence of bilateral pleural effusion, bilateral pulmonary inflammation, cardiac enlargement, and splenic infarction. Furthermore, the electrocardiogram revealed the presence of sinus rhythm and non-specific T-wave changes.

**Figure 1:**
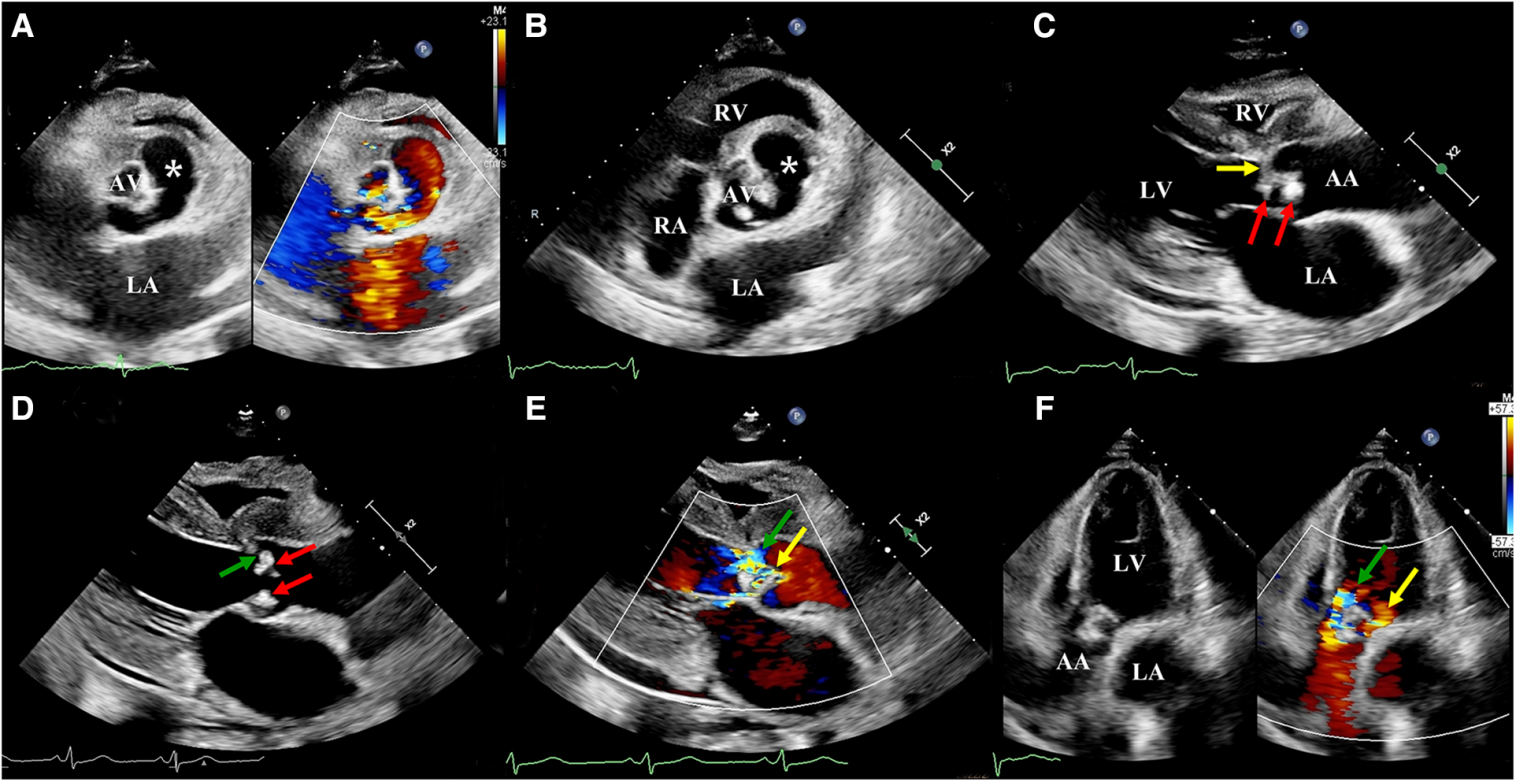
Two-dimensional transthoracic echocardiography: (**A**) A large sac-like aneurysm (white asterisk) located on the left front of the aortic root and communicating with it. (**B**) The number of aortic cusps could not be confirmed, as they were highly calcified and scattered with vegetation. (**C**) Aortic valve scattered with vegetations (red arrow) and local leaflets prolapsed (yellow arrow). (**D**) Aortic valve scattered with vegetation (red arrow) and local leaflet perforated (green arrow). (**E**) Parasternal long-axis view, color Doppler revealed two main aortic regurgitant jets, indicative of perforation (green arrow) and prolapsus (yellow arrow). (**F**) Apical five-chamber view, color Doppler revealed two main aortic regurgitant jets, indicative of perforation (green arrow) and prolapsus (yellow arrow). LA, left atrium; LV, left ventricle; RV, right ventricle; AA ascending aorta.

As the diagnosis of IE was suspected, the patient was followed-up with an ultrasonic examination. Two-dimensional transthoracic echocardiography (2D-TTE) was then performed on an EPIQ CVx cardiac ultrasound system (Epiq CVX, Philips Medical Systems, Andover, MA, United States) with a S9-2 (2–9 MHz) probe. The results illustrated a large aneurysm with the sac-like structure in the left frontal aortic root and communicated with it (Figure 1A). However, the number of aortic cusps could not be confirmed in the short-axis view, as it was highly calcified and scattered with vegetations, leading to mild stenosis (peak systolic velocity: 2.9 m/s) (Figure 1B). In addition, we identified a local prolapse and perforation of the aortic valve with a moderate regurgitation by multibeam (Figures 1C–F). Meanwhile, there was little pericardial effusion. In addition, left ventricular end-diastolic diameter (64 mm) and left ventricular end-systolic diameter (41 mm) revealed dilatation of the left ventricle. The left ventricular ejection fraction was calculated to be 48%. Moreover, tricuspid regurgitation was mild with a pressure gradient of 43 mmHg across the tricuspid valve. There were no other valvular pathologies or other anomalies.

**Figure 2:**
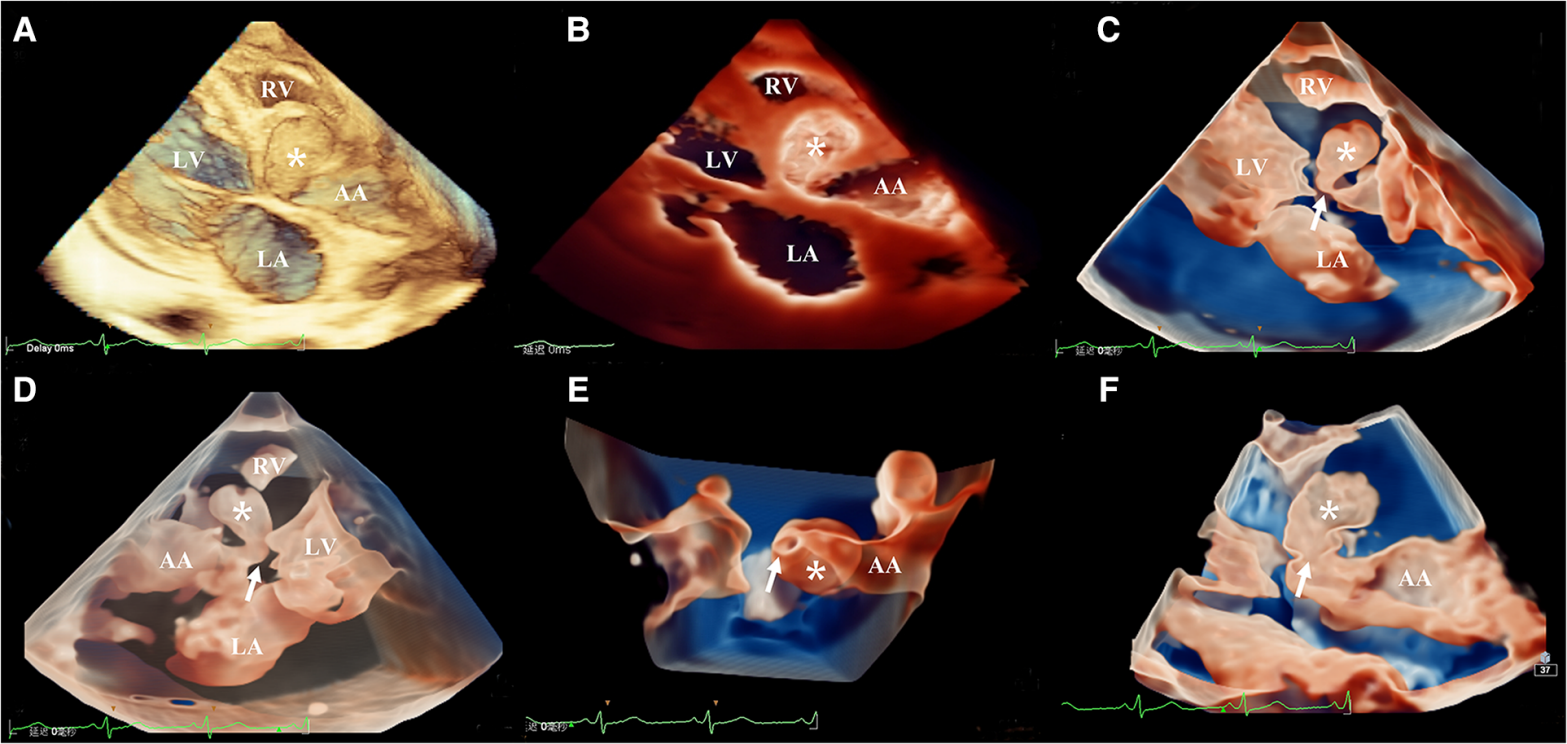
Head-to-head comparisons among traditional three-dimensional transthoracic echocardiography (**A**), TrueVue (**B**), and TrueVue Glass (**C–F**) in the evaluation of the aortic root pseudoaneurysm (white asterisk, its neck: white arrow). Different views of pseudoaneurysm in the TrueVue Glass mode: en face (**C**), dorsal (**D**), bottom (**E**), and internal (**F**) perspective. TrueVue Glass increased the degree of tissue transparency, providing a translucent view, and allowing one to visualize the full view of pseudoaneurysm, as well as its neck. LA, left atrium; LV, left ventricle; RV, right ventricle; AA ascending aorta.

**Figure 3:**
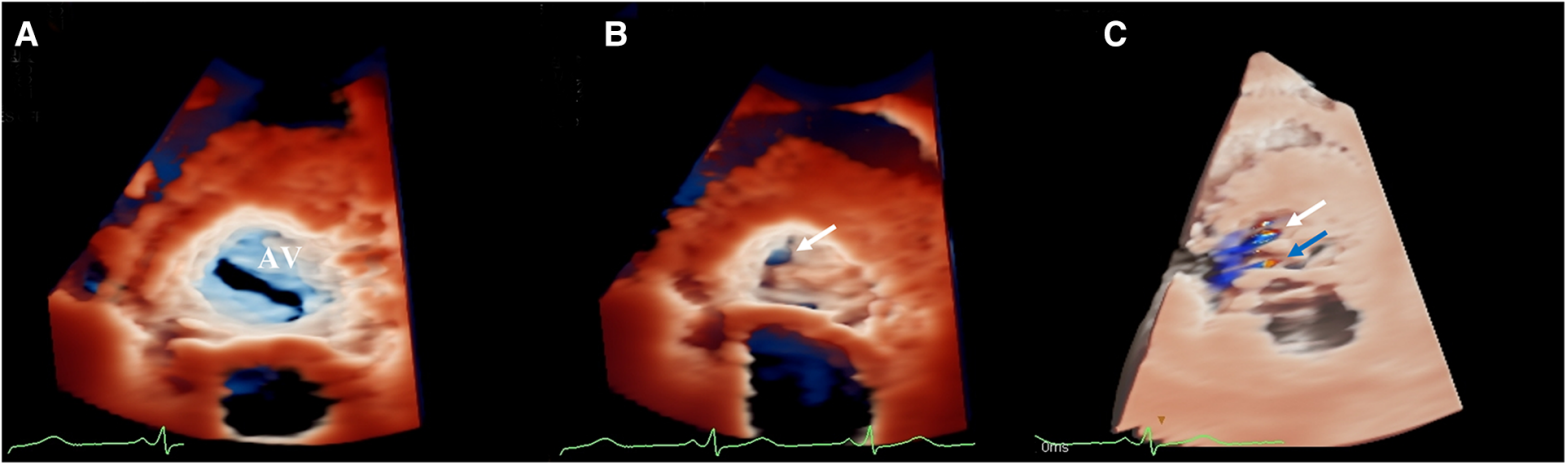
(**A**) TrueVue vividly revealed a bicuspid aortic valve. (**B**) TrueVue clearly showed an echolucent area at the ten o’clock position of the left anterior leaflet, suggesting an area of perforation of 0.24 cm^2^ (white arrow). (**C**) TrueVue merged with three-dimensional color Doppler clearly demonstrated the origin of two different aortic regurgitant jets. One originated from the perforation (white arrow), and the other originated from the prolapsus (blue arrow). AV, aortic valve.

The three-dimensional transthoracic echocardiography (3D-TTE) was performed using an X5-1 (1–5 MHz) probe because the 2D-TTE cannot provide sufficient information. Based on the traditional 3DE imaging technology (Figure 2A), we launched the new TrueVue imaging mode. The virtual light source was introduced and placed inside the aneurysm, which transmitted light from the inside out, improving the border definition (Figure 2B). Next, we initiated the Glass mode to obtain a transparent rendering effect for further evaluation (Figures 2C–F). In this mode, we can obtain a 360° display of aneurysmal morphology and deformation in the cardiac cycle with a click, as well as its neck. In addition, we can also cut off the aneurysm to observe its contour. As a whole, the dimensions of the aneurysm and neck were measured to be 3.62 cm × 2.37 cm × 2.60 cm and 0.22 cm^2^ in size, respectively, without obvious deformation or evidence of rupture. In addition, when the light source was strategically placed behind the aortic valve, the TrueVue imaging mode vividly revealed a bicuspid aortic valve with two leaflets in the left anterior and the right posterior directions, and a straight-line aortic valve closure shape (Figure 3A). The left anterior leaflet prolapsed with a perforation of 0.24 cm^2^ (Figures 3B,C).

In summary, the diagnosis of aortic valve endocarditis was suspected on the basis of characteristic echocardiographic evidence and overall clinical presentation, leading to aortic valve perforation, and prolapse, and aortic root pseudoaneurysm. Antibiotic therapy was initiated immediately with the combined use of vancomycin and gentamycin. Unfortunately, the patient's family refused to provide consent documentation for additional surgery. Despite the active medical treatment modalities, the patient eventually suffered from a sudden, unexpected death 5 days later. Given the lack of family consent, we did not perform an autopsy.

## Discussion

In accordance with the modified Duke criteria, the case reported in the current study fulfilled one major criterion (echocardiography findings) and two minor criterions (fever >38°C and splenic infarction) for inclusion in the study. The diagnosis would at best be classified as “possible” IE (6). The Duke criteria are known for its emphasis on the positive blood cultures, along with echocardiography findings. However, the sensitivity of the criteria for the diagnosis of BCNIE is ambiguous (7). BCNIE refers to IE in which no causative microorganism can be grown employing routine blood culture methods (8). Accordingly, a complete clinical examination is warranted for detection of any accessible infectious focus, which can lead to an early diagnosis in patients with suspected endocarditis, and sometimes can be more effective than blood cultures. Similarly, in our case, the diagnosis of IE benefited from a series of novel three-dimensional echocardiographic methods. Usually, BCNIE, a consequence of previous antibacterial treatment, is commonly precipitated by gram-positive cocci, such as Staphylococci, Streptococci, and Enterococci (9). Other causes include endocarditis related to fastidious, also referred to as the “HACEK” group and the intracellular bacteria that cannot be routinely cultured in blood using the currently available techniques (10). The patient in our study received repeated, inadequate antibiotic treatments at other medical institutions prior to admittance to our Department of Cardiovascular Disease, which may constitute the primary reason for the negative blood culture.

A study revealed that patients with BCNIE present with a higher rate of complications, such as valve rupture and perforation, requiring immediate surgical intervention, compared with those with blood culture-positive endocarditis (BCPIE) (11). Pseudoaneurysm is a rare complication of IE yet possesses a high risk of mortality with inadvertent rupture; accordingly, it requires prompt identification and correct diagnosis (12). Given the typical echocardiographic findings, the patient was indicated for surgery. However, the patient's family was hesitant to proceed with the operation and did not provide additional consent documentation for surgery. Unfortunately, this patient suffered from a sudden unexpected death on the 5th day after the echocardiographic diagnosis. In addition, a previous study conducted by EURO-ENDO registry has indicated an increase of the short-term and long-term mortality in patients with BCNIE relative to those with BCPIE, whereas reduced mortality can be achieved through surgery. This eventually emphasizes that additional efforts are warranted both to improve the etiological diagnosis of IE and to identify BCNIE cases in a timely manner before progressive disease potentially contraindicates surgery (13).

It is well-established that echocardiography plays a crucial role in the diagnosis of IE. Although two-dimensional echocardiography (2DE) and traditional 3DE can provide some diagnostic information, their disadvantages are obvious. The 2DE cannot tell the spatial structure characteristics directly and needed multiple ultrasound sections to clarify the anatomical definition, which was time-consuming, and the diagnosis depended more on the experience of imaging readers, which was not conducive to the care of emergent patients. The image derived from traditional 3DE is usually not clear enough and does not consistently provide adequate detail information or depth perception. Taking the aneurysmal neck as an example, there were certain difficulties for traditional 3DE to display it plainly, due to its smaller and thinner structure. In comparation, the photo-realistic techniques with a higher resolution allows for more realistic images of cardiac structures, by superimposing a virtual light source, which can be moved around and through the aneurysm, making realistic light and shadow effects to highlight the target structures, augmenting our understanding of the anatomical morphology. Studies have shown that novel 3DE rendering techniques can better delineate borders, orifices, cavities, and other structural abnormalities compared with 2DE and traditional 3DE, increasing the diagnostic confidence of readers and improving communication between the surgeon and the imaging specialist (4, 14, 15). To the best of our knowledge, this is the first case diagnosed with BCNIE with aortic valve involvement, leading to aortic valve perforation, and prolapse and development into a giant aortic root pseudoaneurysm using a series of novel three-dimensional echocardiographic methods. In the past, the description of pseudoaneurysms was primarily dependent on the CT. Relative to CT, novel 3D rendering techniques are more convenient and faster, especially the TrueVue Glass mode, which allows for an all-around display of an aortic root pseudoaneurysm with a click. In addition, the region of interest of images can be directly zoomed and rotated with two fingers on the touch screen. More importantly, given the fact that some structures are very mobile, real-time imaging with the help of 3DE can often visualize these structures better than cardiac CT or magnetic resonance imaging. Therefore, with the rapid development of novel 3D rendering techniques, the diagnosis of structural heart diseases will become much more efficacious in the coming years, continuously improving the prognosis and management of such patients.

## Conclusion

BCNIE with aortic valve involvement and development into a giant aortic root pseudoaneurysm is a rare but serious cardiac disease. The case included in the current study endorsed 3D transillumination rendering techniques for the detection of such structural heart disease.

## Data Availability

The original contributions presented in the study are included in the article/**Supplementary Material**, further inquiries can be directed to the corresponding author.
